# The C-reactive protein-to-albumin ratio as a diagnostic biomarker for rheumatoid arthritis: a cross-sectional NHANES analysis

**DOI:** 10.3389/fmed.2025.1624527

**Published:** 2025-07-18

**Authors:** Fan Yang, Weihua Sang, Yongqing Liu, Jun Wang

**Affiliations:** Department of Orthopedics, Cangzhou Central Hospital, Cangzhou, Hebei, China

**Keywords:** C-reactive protein to albumin ratio, rheumatoid arthritis, NHANES, systemic inflammation, prognostic biomarker

## Abstract

**Background:**

Rheumatoid arthritis (RA) is a chronic inflammatory disorder that leads to joint damage, cartilage and bone destruction, and functional disability. The C-Reactive Protein to Albumin Ratio (CAR), an emerging biomarker reflecting systemic inflammation and nutritional status, has demonstrated prognostic value in various diseases. However, its utility in predicting clinical outcomes in RA patients remains underexplored, warranting further investigation to assess its potential role in disease management and prognosis. This cross-sectional study investigates the potential relationship between CAR and RA in United States adults, develops a clinical prediction model, and validates its effectiveness.

**Objective:**

To investigate the association between the CAR and RA using data from the National Health and Nutrition Examination Survey (NHANES).

**Methods:**

This large-scale, cross-sectional study analyzed data from the NHANES database between 1999 and 2018 (excluding 2011–2014). The CAR was calculated as the ratio of C-reactive protein (CRP) to albumin (ALB) levels. RA status was identified through self-reported questionnaire data. Weighted multivariate regression models and subgroup analyses were used to examine the association between CAR and RA. Restricted cubic splines (RCS) were employed to evaluate potential non-linear relationships, and sensitivity analyses were conducted to assess the robustness of the results. Least absolute shrinkage and selection operator (LASSO) were utilized for variable selection in the prediction model. Decision curve analysis (DCA) and receiver operating characteristic (ROC) curve analysis were applied to assess the predictive performance of the models.

**Results:**

This study included a total of 20,733 patients, among whom 1,744 individuals (4.95%) were diagnosed with RA. After controlling for all covariates, the results of multivariate logistic regression analysis indicated a statistically significant correlation between higher Ln(CAR) levels and the increased incidence of RA (OR:1.77 (95% CI, 1.39–2.25); *p* < 0.001). The interaction test results showed that there was no statistically significant influence in this specific association. RCS regression modeling demonstrated a linear relationship between Ln-CAR and RA risk. After variable screening, we constructed an RA prediction model incorporating CAR, and the results were visualized using a nomogram. The area under the curve (AUC) was 0.749 (95% CI, 0.738–0.760), and DCA indicated that the model holds clinical significance.

**Conclusion:**

These findings suggest that CAR may serve as a promising inflammatory biomarker for predicting the presence of RA. In the RA prediction model incorporating CAR, we validated the effectiveness and clinical utility of this model, providing evidence that CAR can serve as a biomarker for RA risk prediction. Further prospective studies are warranted to validate its clinical utility in RA risk stratification and management.

## 1 Introduction

Rheumatoid arthritis (RA) is a prototypical autoimmune condition characterized by persistent, symmetric inflammatory polyarthritis with a predilection for small joints. It frequently leads to bony destruction and is associated with significant systemic morbidity. The pathological changes include synovitis and vasculitis, which gradually destroy articular cartilage and bone, resulting in joint deformity and loss of function (1). Despite decades of in-depth research, RA’s exact etiology and pathogenesis remain unresolved. RA is primarily attributed to the combined effects of genetic susceptibility, environmental factors, and immune system dysregulation (2). Of note, RA poses significant health and socioeconomic challenges to individual patients and healthcare systems. The personal burden is reflected in musculoskeletal impairment, leading to reduced physical function and quality of life, while the societal burden arises from patients’ functional disabilities and the resulting decline in their work capacity (3–5).

The clinical presentation of RA varies significantly between the early and advanced stages. Early RA is typically characterized by systemic symptoms such as morning stiffness, fatigue, joint swelling, and elevated levels of CRP and ESR (6). In contrast, advanced RA is marked by more severe systemic manifestations, including pleural effusion, lymphoma, hematological abnormalities, and cartilage destruction (1, 7, 8). Therefore, early diagnosis and treatment are of great importance.

Systemic inflammation is a hallmark of chronic disease progression. Inflammatory biomarkers include a range of parameters, such as cytokines and acute-phase proteins. Specific immune cell subsets such as platelets, lymphocytes, neutrophils, and monocytes are also involved in disease progression. In addition, inflammatory indices like the neutrophil-to-platelet ratio (NPR) and lymphocyte-to-monocyte ratio (LMR) are increasingly recognized as clinically relevant markers. Three inflammatory indices have been derived: the systemic immune-inflammation index (SII) and the aggregate index of systemic inflammation (AISI). A meta-analysis reported that applying the NLR and PLR can effectively distinguish disease severity in RA patients (9). A recent study by Liu et al. examined the association between SII and RA development, demonstrating a statistically significant positive correlation between elevated SII levels and increased RA risk (10).

The C-reactive protein-to-albumin ratio (CAR), derived from CRP and albumin (ALB) levels (CRP/ALB), has emerged as a promising inflammatory biomarker. CRP is a reactive substance that increases during the acute phase of inflammation, whereas albumin is a reactive substance that decreases in concentration during the same phase (11, 12). Emerging evidence highlights the broad clinical utility of CAR as a robust inflammation-related biomarker, demonstrating prognostic value across various pathological conditions, including cardiovascular, cerebrovascular, oncologic, and infectious diseases (13–16). Previous studies exploring the relationship between CAR and RA have been limited to single-center investigations with small sample sizes (17, 18). Besides, these studies lack representativeness and generalizability. To that end, we initiated the first nationwide large-sample clinical study to investigate this relationship further. Additionally, the performance of CAR as a predictive model for RA was evaluated.

## 2 Materials and methods

### 2.1 Study population

The National Health and Nutrition Examination Survey (NHANES) is a nationally representative, cross-sectional surveillance program conducted by the Centers for Disease Control and Prevention (CDC) to systematically assess the health and nutritional status of the United States population. As the only national survey that combines comprehensive health interviews, standardized physical examinations, and detailed laboratory testing across all age groups, the NHANES database offers a unique and robust data source for population health surveillance. The survey collects multidimensional data, including sociodemographic characteristics, self-reported health behaviors and conditions, clinically validated physical measurements, and objective biomarkers from biological specimens, thereby supporting rigorous epidemiological research and evidence-based policy development.

The present study utilized data from 10 consecutive National Health and NHANES cycles, covering the period from 1999 to 2018. To maintain consistency in the availability of key exposure variables, data from the 2011–2014 cycles were excluded, as these did not include the specific exposure measures required for our analysis. The remaining 8 cycles (1999–2010 and 2015–2018) provided a robust, nationally representative sample for our investigation, allowing for a comprehensive evaluation of trends and associations over nearly two decades.

The National Health and NHANES, administered by the National Center for Health Statistics (NCHS) of the CDC, is a nationally representative survey of the United States civilian, non-institutionalized population. Approved by the NCHS Research Ethics Review Board, NHANES collects data with informed written consent from all participants. The datasets used in this study are publicly available on the NHANES website^1^.

**FIGURE 1:**
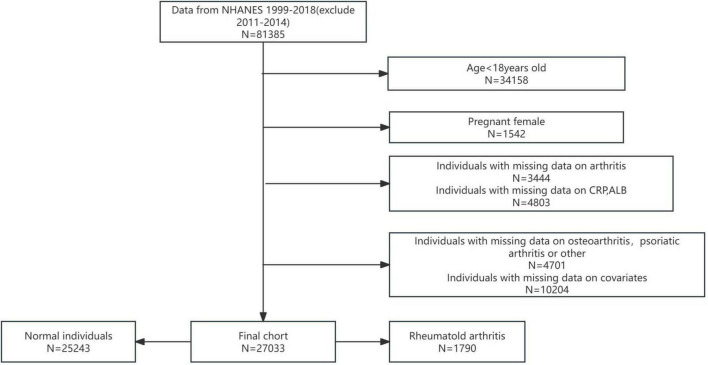
Participant selection flowchart.

In this study, rigorous exclusion criteria were applied to ensure the integrity and validity of the analysis. Participants were excluded based on the following criteria: (i) age under 18 years; (ii) pregnancy; (iii) incomplete or missing data on arthritis status; (iv) missing data on CRP or ALB levels; (v) missing data on osteoarthritis (OA), physical activity (PA), or other critical variables; and (vi) missing data on relevant covariates (Figure 1). After applying these criteria, a final analytical cohort of 27,033 participants was established.

To account for the complex survey design and ensure nationally representative estimates, Mobile Examination Center (MEC) weights were applied in accordance with NHANES analytical protocols. For the 1999–2000 and 2001–2002 cycles, weights were calculated using the formula 2/8 × wtmec4yr, while for the 2003–2010 and 2015–2018 cycles, the formula 1/8 × wtmec2yr was used. This methodological approach is consistent with established NHANES guidelines and enhances the generalizability and reliability of the study findings.

### 2.2 C-reactive protein to albumin ratio

Herein, the CAR was calculated as the ratio of CRP concentration (mg/L) to ALB level (g/L). CRP and ALB measurements were obtained from blood samples collected at the MEC and subsequently analyzed in a central laboratory. Prior to collection, blood specimens were screened using predefined exclusion criteria to ensure data quality. NHANES also implemented rigorous quality control procedures throughout the collection and laboratory testing processes to ensure the accuracy and reliability of results. This standardized approach enhances the validity of CAR as a biomarker in our analysis. For regression analysis, the CAR variable underwent natural logarithmic (Ln) transformation to address the right-skewed distribution of inflammatory indices. This transformation improved data normality and strengthened the robustness of the statistical models.

### 2.3 Definition of rheumatoid arthritis

Arthritis diagnosis was assessed using a validated self-report instrument (MCQ160a), which employed a binary response format (yes/no) to determine whether participants had received a clinician-confirmed arthritis diagnosis. For those who answered affirmatively, subsequent items identified the disease subtype through categorical options: “RA,” “Osteoarthritis,” “Psoriatic arthritis,” “Other,” “Refused,” and “Do not know.” This approach is consistent with previous validation studies demonstrating 85% concordance between self-reported arthritis diagnoses and clinical records, supporting its methodological validity for epidemiological surveillance in this cohort (19).

### 2.4 Covariates

For this study, we extracted comprehensive data on various demographic, socioeconomic, lifestyle, and clinical factors. Demographic and clinical covariates included self-reported sex, age, race/ethnicity (categorized as Mexican American, Other Hispanic, Non-Hispanic Black, Non-Hispanic White, or Other Race), and education level. Anthropometric measures included body mass index (BMI), stratified using ≤ 25.0 kg/m^2^, 25.0–30.0 kg/m^2^, and > 30.0 kg/m^2^. Socioeconomic status was assessed using the poverty-to-income ratio (PIR), categorized as <1.3, 1.3–3.5, and > 3.5. Lifestyle factors consisted of self-reported smoking history (defined as having smoked ≥100 cigarettes in a lifetime) and alcohol consumption in the past 12 months. Comorbidities included hypertension and diabetes. Laboratory parameters comprised complete blood count indices, focusing on CRP and ALB levels. These variables were selected to enable a multidimensional assessment of participant characteristics and health status, ensuring a robust analysis of the relationships under investigation.

### 2.5 Data analysis techniques

All analyses were conducted using R statistical software (version 4.2.2; R Foundation for Statistical Computing). To account for the complex, multistage probability sampling design of the NHANES, we applied appropriate examination weights (wtmec4yr for combined cycles 1999–2002 and wtmec2yr for individual cycles) in all analyses. Continuous variables are presented as weighted means with standard deviations, while categorical variables are expressed as weighted percentages. Between-group comparisons were performed using design-adjusted χ^2^ tests for categorical variables and weighted Student’s *t*-tests for continuous variables.

Given the right-skewed distribution of the CAR values, we performed a logarithmic transformation prior to analysis. Weighted multivariable logistic regression models were constructed to examine the association between RA and CAR across three progressively adjusted models: Crude Model: unadjusted analysis; Model 1: adjusted for demographic factors (age, sex, race/ethnicity); Model 2: further adjusted for socioeconomic status (PIR, education level) and BMI; and Model 3: additional adjustment for clinical covariates (diabetes mellitus, hypertension status) and behavioral factors (alcohol consumption, smoking status). To ensure the robustness of our findings, we conducted sensitivity analyses using complete-case analysis and unweighted multivariable logistic regression to evaluate potential sampling design effects. All analyses accounted for the NHANES complex survey design and incorporated appropriate sampling weights.

To evaluate the non-linear association between Ln(CAR) and RA risk, we categorized Ln(CAR) into quartile-based strata and applied restricted cubic splines (RCS) for flexible modeling of dose-response relationships. Advanced analytical techniques, including generalized additive models (GAMs) with smooth curve fitting, were used to investigate potential threshold effects and identify the inflection point where the risk association pattern changes. To assess potential effect modification, we performed interaction testing and stratified analyses across key sociodemographic and clinical factors, including age, PIR, BMI, race, education, diabetes status, hypertension, alcohol consumption, and smoking behavior. Statistical significance was defined as *p* < 0.05, with rigorous adjustment for multiple comparisons to control for Type I error. This comprehensive analytical approach allowed for the examination of heterogeneity in Ln(CAR)-RA associations across diverse population subgroups.

Variable selection was performed using least absolute shrinkage and selection operator (LASSO) regression with 10-fold cross-validation to determine the optimal penalty parameter (λ). Model discrimination was evaluated through receiver operating characteristic (ROC) curve analysis, with performance quantified by the area under the curve (AUC). We further assessed clinical utility using decision curve analysis (DCA) to estimate the net benefit across a range of probability thresholds.

The final prediction model was presented as a nomogram, which visually displays the weighted contribution of each predictor variable and enables calculation of individualized RA risk probabilities. Points are assigned for each risk factor based on its regression coefficient, with the sum of points corresponding to a predicted probability of RA development.

## 3 Results

### 3.1 Population parameters

From Table 1: this study included 27,033 participants, consisting of 14,017 males (51.39%) and 12,926 females (48.61%). Of these, 18,674 individuals (79.93%) were under 60 years old, while 8,359 individuals (20.07%) were over 60 years old. The racial composition was as follows: 12,294 White People (69.60%), 540 Black People (10.59%), 5,351 Mexican Americans (8.33%), and 3,984 individuals from other races (11.48%). The study focused on four groups based on logarithm-transformed C-reactive protein-to-albumin ratio (LnCAR), with RA present in 239 individuals (2.69%) in Q1, 3,729 individuals (4.10%) in Q2, 488 individuals (5.78%) in Q3, and 645 individuals (7.78%) in Q4.

**TABLE 1:** Population characteristics by categories of CAR by sampling weight.

Characteristic	Total (*n* = 27033)	Q1 (*n* = 6743)	Q2 (*n* = 6741)	Q3 (*n* = 6777)	Q4 (*n* = 6772)	*p*-value^1^
Gender						< 0.001
Male	14,107 (51.39%)	3,935 (56.31%)	4,018 (59.12%)	3,461 (50.54%)	2,693 (37.49%)	
Female	12,926 (48.61%)	2,808 (43.69%)	2,723 (40.88%)	3,316 (49.46%)	4,079 (62.51%)	
Age, years						< 0.001
<60	18,674 (79.93%)	5,292 (86.38%)	4,611 (78.81%)	4,378 (76.47%)	4,393 (76.81%)	
≥ 60	8,359 (20.07%)	1,451 (13.62%)	2,130 (21.19%)	2,399 (23.53%)	2,379 (23.19%)	
Race						< 0.001
Non-Hispanic White	12,294 (69.60%)	3,273 (72.03%)	3,137 (70.72%)	3,047 (68.62%)	2,837 (66.33%)	
Non-Hispanic Black	5,404 (10.59%)	1,161 (8.62%)	1,213 (9.34%)	1,325 (10.66%)	1,705 (14.35%)	
Mexican American	5,351 (8.33%)	1,119 (6.80%)	1,365 (8.84%)	1,451 (8.86%)	1,416 (9.12%)	
Other	3,984 (11.48%)	1,190 (12.55%)	1,026 (11.09%)	954 (11.86%)	814 (10.20%)	
Education						< 0.001
<9	3,314 (5.51%)	612 (4.08%)	863 (5.91%)	936 (6.10%)	903 (6.19%)	
09–12	10,385 (35.88%)	2,307 (30.39%)	2,540 (35.05%)	2,699 (38.74%)	2,839 (40.62%)	
>12	13,334 (58.61%)	3,824 (65.53%)	3,338 (59.04%)	3,142 (55.16%)	3,030 (53.18%)	
Family income						< 0.001
≤1.3	7,933 (20.02%)	1,739 (17.05%)	1,801 (18.17%)	2,044 (21.08%)	2,349 (24.65%)	
1.3–3.5	10,543 (36.06%)	2,504 (33.87%)	2,691 (36.00%)	2,705 (37.64%)	2,643 (37.19%)	
>3.5	8,557 (43.92%)	2,500 (49.08%)	2,249 (45.83%)	2,028 (41.28%)	1,780 (38.16%)	
BMI, kg/m^2^						< 0.001
<25	8,149 (32.55%)	3,724 (57.35%)	2,076 (32.15%)	1,350 (20.21%)	999 (15.21%)	
25–30	9,361 (33.66%)	2,249 (31.85%)	2,859 (41.87%)	2,466 (34.65%)	1,787 (25.66%)	
>30	9,523 (33.79%)	770 (10.81%)	1,806 (25.97%)	2,961 (45.14%)	3,986 (59.13%)	
Alcohol						< 0.001
No	7,186 (22.20%)	1,568 (19.17%)	1,574 (19.38%)	1,863 (23.00%)	2,181 (28.30%)	
Yes	19,847 (77.80%)	5,175 (80.83%)	5,167 (80.62%)	4,914 (77.00%)	4,591 (71.70%)	
Diabetes						< 0.001
No	18,458 (73.17%)	5,383 (83.34%)	4,760 (74.92%)	4,360 (69.10%)	3,955 (62.87%)	
Yes	8,575 (26.83%)	1,360 (16.66%)	1,981 (25.08%)	2,417 (30.90%)	2,817 (37.13%)	
Hypertension						< 0.001
No	24,069 (92.56%)	6,307 (96.08%)	6,094 (93.93%)	5,968 (91.12%)	5,700 (88.18%)	
Yes	2,964 (7.44%)	436 (3.92%)	647 (6.07%)	809 (8.88%)	1,072 (11.82%)	
Smoke						< 0.001
No	14,451 (53.41%)	3,909 (58.27%)	3,621 (53.65%)	3,459 (50.02%)	3,462 (50.70%)	
Yes	12,582 (46.59%)	2,834 (41.73%)	3,120 (46.35%)	3,318 (49.98%)	3,310 (49.30%)	
CRP, mg/l						< 0.001
Median (IQR)	1.80 (0.70, 4.10)	0.49 (0.30, 0.60)	1.30 (1.00, 1.60)	2.90 (2.40, 3.60)	7.70 (5.70, 11.90)	
ALB, g/l						< 0.001
Median (IQR)	43.00 (41.00, 45.00)	44.00 (43.00, 46.00)	43.00 (42.00, 46.00)	43.00 (41.00, 45.00)	41.00 (39.00, 43.00)	
RA						< 0.001
No	24,748 (95.05%)	6,288 (97.31%)	6,342 (95.90%)	6,140 (94.22%)	5,978 (92.22%)	
Yes	1,744 (4.95%)	239 (2.69%)	372 (4.10%)	488 (5.78%)	645 (7.78%)	

^1^chi-squared test with Rao & Scott’s second-order correction; Wilcoxon rank-sum test for complex survey samples.

### 3.2 Correlation between RA and CAR

The results of the weighted multivariate logistic regression analysis demonstrate a strong association between an elevated CAR and an increased risk of developing RA (Table 2). This association remained consistent across sequentially adjusted models. In the unadjusted model, each unit increase in Ln(CAR) was associated with a 40.1% higher odds of RA (odds ratio [OR] 1.41; 95% CI, 1.34–1.48; *P* < 0.001). After adjusting for sex, age, and race in model 1, the effect estimate remained significant (OR 1.34; 95% CI, 1.27–1.41; *P* < 0.001). Model 2, which incorporated additional covariates such as PIR, BMI, and education level, yielded a modestly attenuated but still significant association (OR 1.21; 95% CI, 1.14–1.28; *P* < 0.001).

**TABLE 2:** Weighted multivariate logistic analysis.

Variable	Crude		Model 1		Model 2		Model 3	
	OR (95% CI)	*P*	OR (95% CI)	*P*	OR (95% CI)	*P*	OR (95% CI)	P
LNCAR	1.41 (1.34, 1.48)	< 0.001	1.34 (1.27, 1.41)	< 0.001	1.26 (1.19, 1.33)	< 0.001	1.21 (1.14, 1.28)	< 0.001
**Stratified by LNCAR quartiles**								
Q1	1 (Ref)		1 (Ref)		1 (Ref)		1 (Ref)	
Q2	1.59 (1.22, 2.08)	< 0.001	1.4 (1.07, 1.84)	0.015	1.3 (0.99, 1.71)	0.063	1.24 (0.95, 1.64)	0.118
Q3	2.31 (1.81, 2.95)	< 0.001	1.93 (1.50, 2.47)	< 0.001	1.63 (1.26, 2.12)	< 0.001	1.51 (1.16, 1.96)	0.003
Q4	3.13 (2.51, 3.91)	< 0.001	2.54 (2.02, 3.18)	< 0.001	2.01 (1.59, 2.56)	< 0.001	1.77 (1.39, 2.25)	< 0.001
P for trend		< 0.001		< 0.001		< 0.001		< 0.001

Crude: unadjusted analysis. Model 1: the variables of age, gender, and race were subjected to adjustment. Model 2: the variables of gender, age, race, PIR, education, and BMI were subjected to adjustment. Model 3: the variables of gender, age, race, PIR, education, BMI, diabetes, hypertension, alcohol consumption, smoke habits were subjected to adjustment.

To evaluate the sensitivity of these findings and explore potential non-linear relationships, Ln(CAR) was analyzed as a categorical variable stratified by quartiles. Compared with the lowest quartile (Q1), individuals in the highest quartile (Q4) exhibited a 213% increase in the odds of RA in the unadjusted analysis (odds ratio [OR] 3.13; 95% CI, 2.51–3.91; *P* < 0.001). This elevated risk remained significant in Model 1 (OR 2.54; 95% CI, 2.02–3.18; *P* < 0.001) and Model 2 (OR 2.01; 95% CI, 1.59–2.56; *P* < 0.001), with a gradual attenuation of effect size as covariates were added. In the fully adjusted Model 3, the highest quartile of Ln(CAR) still conferred a 77% higher odds of RA compared with the lowest quartile (OR 1.77; 95% CI, 1.39–2.25; *P* < 0.001), highlighting the independent prognostic value of CAR in RA risk stratification.

To further assess the consistency of the CAR-RA association and the potential impact of weighting methods, we conducted sensitivity analyses using an unweighted approach (Table 3). These additional analyses confirmed the primary findings, showing a consistent positive relationship between elevated CAR and RA risk. In all models, from unadjusted to fully adjusted, the direction and statistical significance of this association remained consistent, with effect sizes similar to those in the weighted analysis. These results reinforce the robustness of our primary findings, establishing CAR as a reliable biomarker for RA risk stratification, regardless of the analytical weighting method used. The consistency across different methodological approaches further supports the biological plausibility and clinical relevance of the CAR-RA relationship.

**TABLE 3:** Sensitivity analysis.

Variable	Crude		Model 1		Model 2		Model 3	
	OR (95% CI)	*P*	OR (95% CI)	*P*	OR (95% CI)	*P*	OR (95% CI)	*P*
LNCAR	1.37 (1.32 ∼ 1.42)	< 0.001	1.28 (1.23 ∼ 1.33)	< 0.001	1.2 (1.15 ∼ 1.26)	< 0.001	1.17 (1.12 ∼ 1.22)	< 0.001
**Stratified by LNCAR quartiles**								
Q1	1 (Ref)		1 (Ref)		1 (Ref)		1 (Ref)	
Q2	1.58 (1.34 ∼ 1.87)	< 0.001	1.36 (1.15 ∼ 1.6)	< 0.001	1.25 (1.06 ∼ 1.49)	0.009	1.22 (1.03 ∼ 1.44)	0.025
Q3	2.15 (1.84 ∼ 2.52)	< 0.001	1.71 (1.46 ∼ 2.01)	< 0.001	1.47 (1.24 ∼ 1.73)	< 0.001	1.39 (1.18 ∼ 1.64)	< 0.001
Q4	2.95 (2.54 ∼ 3.43)	< 0.001	2.24 (1.92 ∼ 2.62)	< 0.001	1.79 (1.52 ∼ 2.11)	< 0.001	1.63 (1.38 ∼ 1.93)	< 0.001
P for trend		< 0.001		< 0.001		< 0.001		< 0.001

Crude: unadjusted analysis. Model 1: the variables of age, gender, and race were subjected to adjustment. Model 2: the variables of gender, age, race, PIR, education, and BMI were subjected to adjustment. Model 3: the variables of gender, age, race, PIR, education, BMI, diabetes, hypertension, alcohol consumption, smoke habits were subjected to adjustment.

Crude: unadjusted analysis.
Model 1 included baseline demographic adjustments for age, sex, and self-reported race/ethnicity.
Model 2 built upon Model 1 by incorporating socioeconomic and lifestyle factors, including PIR, educational attainment, and BMI.
Model 3 represented the fully adjusted specification, which further accounted for clinical comorbidities and behavioral covariates, such as diabetes status, hypertension diagnosis, alcohol consumption frequency, and smoking history.

Crude: unadjusted analysis.
Model 1 included baseline demographic adjustments for age, sex, and self-reported race/ethnicity.
Model 2 expanded on Model 1 by incorporating socioeconomic and lifestyle factors, including PIR, educational attainment, and BMI.
Model 3 represented the fully adjusted specification, which added clinical comorbidities and behavioral covariates, such as diabetes status, hypertension diagnosis, alcohol consumption frequency, and smoking history.

### 3.3 Linear relationship between RA and CAR

**FIGURE 2:**
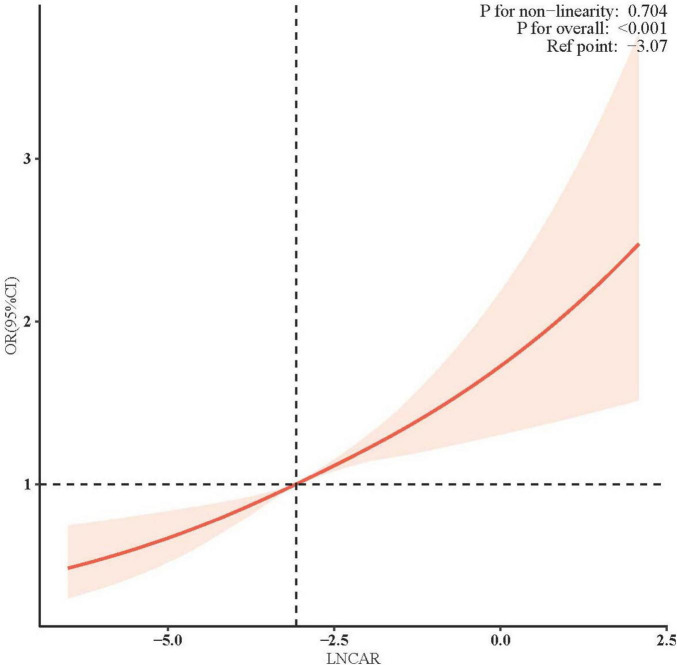
Adjusted association of LnCAR with RA Risk.

Figure 2 illustrates the adjusted association between the natural LnCAR, modeled as a continuous variable, and the risk of incident RA. After comprehensive adjustment for demographic, socioeconomic, lifestyle, and clinical confounders, a clear linear dose-response relationship was observed, with higher LnCAR values corresponding to progressively increased RA risk. Specifically, each one-unit increase in LnCAR was associated with a significant and graded rise in the adjusted odds ratio (OR) for RA, indicating a strong and biologically plausible risk gradient. These findings remained consistent across sensitivity analyses, further supporting the independent role of systemic inflammation, as reflected by CAR, in RA pathogenesis.

Multivariable logistic regression models were constructed with adjustments for key confounders, including sex, age, race/ethnicity, BMI, educational attainment, diabetes status, hypertension, alcohol consumption, and smoking history. The solid red line represents the adjusted OR for RA per one-unit increase in natural LnCAR, while the shaded red area indicates the 95% CI. The overall association was highly significant (*P* < 0.001), and no evidence of non-linearity was observed, as indicated by a non-significant *P*-value (>0.05) from the likelihood ratio test comparing nested linear and non-linear models. These findings support a strong linear dose-response relationship between elevated systemic inflammation, as reflected by CAR, and increased RA risk.

### 3.4 Subgroup analysis

**FIGURE 3:**
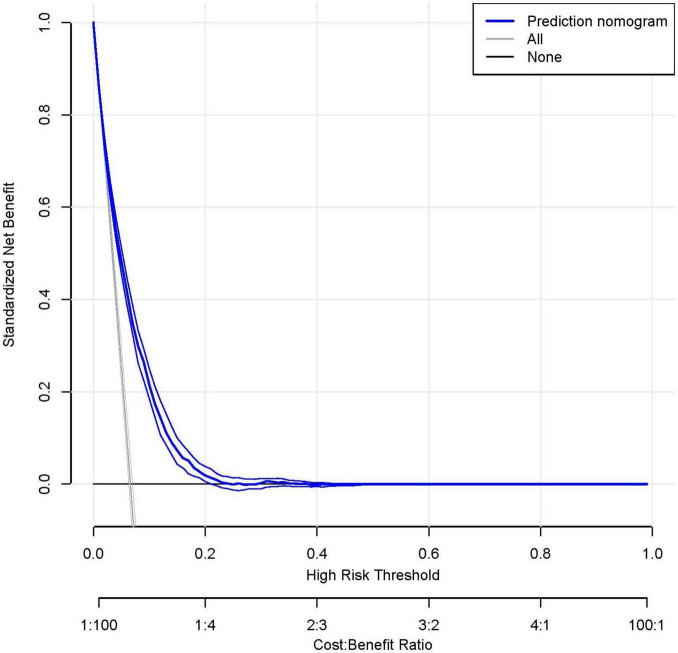
Forest plot for the correlation between CAR and RA.

The results of the interaction test (Figure 3) indicate that age, sex, race, PIR, BMI, hypertension, diabetes, alcohol consumption, and smoking history did not significantly modify the association between CAR and RA risk, as all interaction *P*-values were greater than 0.05.

### 3.5 Variable selection for RA prediction model

Prior to model development, we performed LASSO regression to identify predictive variables. Through 10-fold cross-validation, the optimal regularization parameter (λ = 0.006) was selected to minimize prediction error. Variables with an odds ratio (OR) of 1 (indicating no association) were excluded.

**FIGURE 4:**
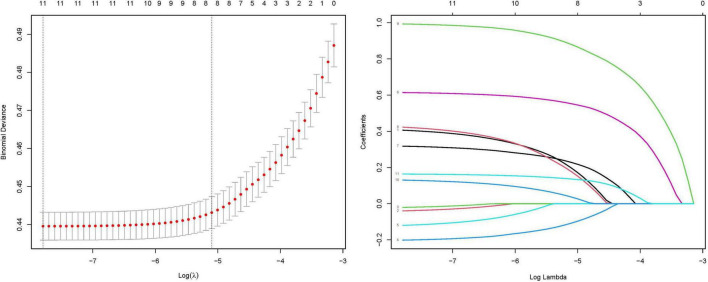
Coefficient profiles of the LASSO regression model. Each trajectory represents the coefficient path of a specific variable as the regularization parameter (λ) varies. Ten-fold cross-validation for tuning parameter (λ) selection in the LASSO model. The red dotted line indicates the optimal λ value that minimizes the mean squared error (MSE), as determined by cross-validation. The left vertical dashed line corresponds to the λ value yielding the most parsimonious model within one standard error of the minimum MSE (1-SE criterion). The numbers along the top axis indicate the count of retained variables at each λ value.

**FIGURE 5:**
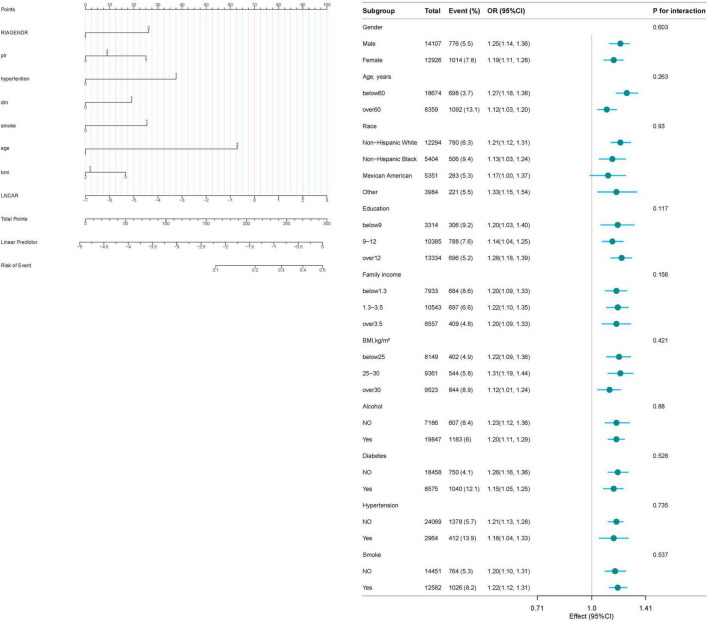
This nomogram provides a clinical tool to estimate an individual’s probability of developing rheumatoid arthritis (RA) based on eight key predictors. Each variable is categorized as follows: sex: 1 = male; 2 = female; platelet-to-lymphocyte ratio (PLR): 1 = ≤ 1.3 (low); 2 = 1.3–3.5 (intermediate); 3 = > 3.5 (high); hypertension: 1 = present; 2 = absent; smoking status: 1 = current or former smoker; 2 = never smoker; age: 1 = ≤ 60 years; 2 = > 60 years; body mass index (BMI): 1 = < 25 kg/m^2^ (normal weight); 2 = 25–30 kg/m^2^ (overweight); 3 = > 30 kg/m^2^ (obese); Diabetes mellitus: 1 = present; 2 = absent; C-reactive protein–albumin ratio (lnCAR): continuous scale (log-transformed).

The final prediction model incorporated eight clinically relevant variables: age, sex, CAR, hypertension, smoking status, platelet-to-lymphocyte ratio (PLR), BMI, and diabetes mellitus. Results of the variable selection process (LASSO coefficient trajectories and cross-validation) are presented in Figure 4, while the final model predictors are detailed in Figure 5.

### 3.6 An evaluation of the predictive capability of the prediction model

**FIGURE 6:**
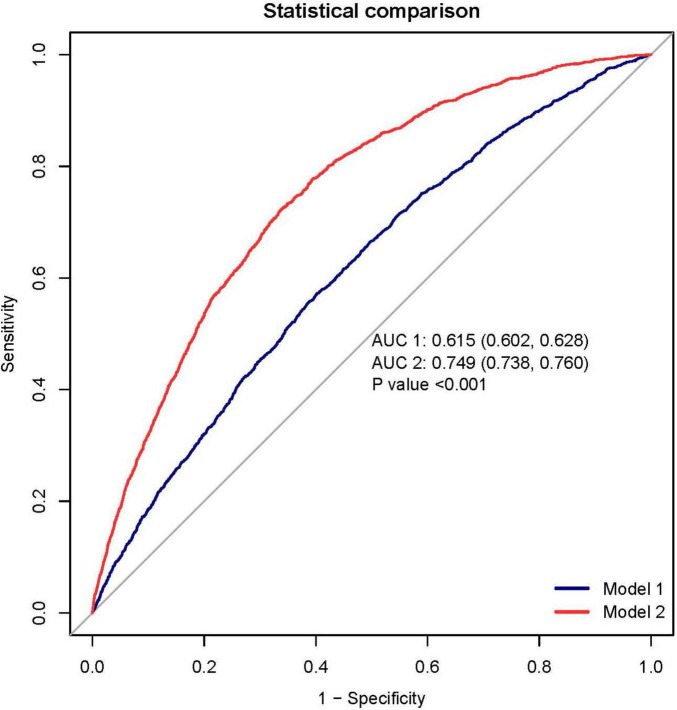
The receiver operating characteristic (ROC) analysis demonstrates superior discrimination of the rheumatoid arthritis (RA) prediction model (red curve; AUC 0.749, 95% CI 0.738–0.760) compared to the C-reactive protein-albumin ratio (CAR) alone (blue curve; AUC 0.615, 95% CI 0.602–0.628). At the optimal cutoff, the prediction model achieved a sensitivity of 72.2% and specificity of 66.3%. Decision curve analysis (DCA) reveals the clinical utility of the prediction model (blue curve), which provides greater net benefit across most threshold probabilities than either the “treat all” or “treat none” strategies (represented by the black and gray reference lines, respectively). This suggests the model’s potential to guide clinical decision-making by more accurately identifying high-risk patients who would benefit from intervention.

To assess the predictive accuracy of the CAR and the RA prediction model, ROC curves were analyzed. The CAR model demonstrated modest discrimination, with an AUC of 0.615 (95% CI, 0.602–0.628). In contrast, the comprehensive prediction model showed significantly improved performance, achieving an AUC of 0.749 (95% CI, 0.738–0.760), with a sensitivity of 72.2% and specificity of 66.3% (Figure 6).

To facilitate clinical implementation, we developed a nomogram (Figure 5) that integrates all predictor variables into an intuitive scoring system. Each variable is weighted according to its contribution to RA risk, and the total score corresponds to a predicted probability of RA development.

Decision curve analysis further validated the model’s clinical value, demonstrating superior net benefit compared to the “treat all” and “treat none” strategies across a clinically relevant range of threshold probabilities (Figure 6). This supports its utility for individualized risk assessment and decision-making in clinical practice.

## 4 Discussion

In this cross-sectional analysis of 20,733 participants from the NHANES database (1999–2018), including 14,107 males and 12,926 females, a total of 1,744 cases of RA were identified. Comparative analysis showed significantly higher CAR levels in individuals with RA than those without the disease (*P* < 0.001). Multivariable-adjusted regression models demonstrated a linear dose-response association between CAR and RA risk, with increasing CAR levels corresponding to a progressive rise in RA prevalence (P for trend < 0.001). Importantly, we found that higher CAR levels were significantly associated with RA activity. The prevalence of RA in the highest CAR quartile was 77% higher than in the lowest quartile, underscoring the potential predictive value of CAR as a marker of disease activity in RA. We developed and rigorously validated a clinical prediction model incorporating the CAR, which demonstrated significant discriminative ability (AUC 0.749) and clinical utility through DCA. Notably, the nomogram revealed that CAR contributed the highest weighted score among all predictive variables, underscoring its dominant role in RA risk stratification.

Rheumatoid arthritis is a chronic autoimmune disease characterized by synovial inflammation and progressive joint destruction. It affects approximately 0.5%–1% of the adult population, with women being three times more likely to develop the disease than men (1). Currently, the diagnosis of RA relies on a combination of clinical symptoms, physical examination findings, standardized questionnaires, and laboratory tests (20). Among laboratory indicators, ESR and CRP are the most commonly used markers for assessing disease activity in clinical practice (21). However, both markers have certain limitations. They primarily reflect short-term inflammatory activity and cannot effectively differentiate RA from other overlapping inflammatory conditions (22–24). Elevations in CRP, fibrinogen, and ferritin, key acute-phase reactants are characteristic of autoimmune diseases such as RA, reflecting systemic inflammation and complementing the inflammatory cascade driven by pro-inflammatory cytokines, particularly interleukin-6 (IL-6). The soluble human leukocyte antigen-G (sHLA-G) molecule, a key immunomodulatory factor, has been implicated in immune-mediated diseases, including RA. As demonstrated by Contini et al. (25), sHLA-G modulates inflammatory responses by regulating T-cell, B-cell, and dendritic cell activity, thereby promoting immune tolerance. Interactions between sHLA-G and inflammatory biomarkers such as CRP may offer novel insights into RA pathogenesis. Notably, sHLA-G may attenuate CRP-driven inflammatory cascades, suggesting a protective role in RA progression. Further investigation into the relationship between sHLA-G and the CAR could clarify its potential as a complementary biomarker for RA activity and prognosis. CAR, defined as the ratio of CRP to ALB, integrates two core biomarkers representing inflammatory and nutritional status (26, 27). CRP, a pentameric protein synthesized in the liver, is upregulated during the acute-phase response. Its production is stimulated by pro-inflammatory cytokines, including interleukin-1 (IL-1), IL-6, and tumor necrosis factor-alpha (TNF-α), making CRP a sensitive marker of systemic inflammation (28). In contrast, albumin, synthesized primarily in the liver, accounts for approximately 60% of total serum proteins (29). Clinically, serum albumin levels are widely used to assess nutritional status, with hypoalbuminemia recognized as a marker of various pathological conditions, including renal dysfunction, hepatic impairment, protein-energy malnutrition, chronic infections, and malignancies (30, 31). Recent studies have shown that CAR serves as a valuable prognostic indicator in inflammatory conditions by reflecting the combined effects of systemic inflammation (via CRP) and nutritional status (via albumin), thereby offering insight into disease severity and outcomes (32, 33). CAR can serve as a predictive indicator of mortality in certain diseases and has been shown to outperform CRP or albumin alone in this regard (34, 35). Sheinenzon et al. found that CRP has minimal influence on albumin levels, with a statistically significant negative correlation observed only at markedly elevated CRP concentrations (≥500 mg/L) (36). Given its greater stability under conditions of extreme inflammation, CAR provides a more reliable assessment of a patient’s combined inflammatory and nutritional status.

Although previous studies have explored the relationship between CAR and RA, for instance, Yang et al. analyzed 319 individuals and identified a correlation between CAR and RA (17), and Kaplan et al. similarly reported this association (18), our study offers several advantages. Unlike these earlier investigations, our analysis is based on a nationally representative sample with a substantially larger population and incorporates both multivariate and sensitivity analyses. These methodological strengths enhance the stability and reliability of our findings, supporting the clinical utility of CAR as a robust indicator for assessing RA activity.

Despite leveraging a nationally representative sample, this study has several limitations. First, the restriction to adults and reliance on self-reported RA diagnoses may introduce misclassification bias, although we applied stringent criteria (e.g., requiring explicit endorsement of RA in NHANES questionnaires) to enhance specificity, consistent with prior NHANES analyses (10). Second, while we adjusted for key covariates, residual confounding (e.g., undetected acute infections or nuanced nutritional deficits) may persist. Third, exclusion of NHANES 2011–2014 data—due to protocol-driven unavailability of CRP measurements—could affect temporal generalizability. To further elucidate the causal relationship between CAR and RA and the biological mechanisms underlying this association, prospective cohort studies are warranted.

## 5 Conclusion

This cross-sectional study demonstrates a significant correlation between CAR and RA in adult patients in the United States. The RA prediction model incorporating CAR demonstrated robust discriminatory performance (AUC 0.749, 95% CI 0.738–0.760) and meaningful clinical utility in risk stratification. These findings position CAR as a promising biomarker for RA prediction, supported by its biological plausibility in reflecting systemic inflammation. However, given the cross-sectional design and reliance on self-reported RA diagnoses, further validation in large, multicenter prospective cohorts is essential to confirm its predictive value and clinical applicability.

## Data Availability

The datasets presented in this study can be found in online repositories. The names of the repository/repositories and accession number(s) can be found in the article/supplementary material.

## Footnotes

1
http://www.cdc.gov/nchs/nhanes/index.htm

## References

[1] Smolen J Aletaha D McInnes I. Rheumatoid arthritis. *Lancet.* (2016) 388:2023–38. 10.1016/S0140-6736(16)30173-8 27156434

[2] McInnes I Schett G. The pathogenesis of rheumatoid arthritis. *N Engl J Med.* (2011) 365:2205–19. 10.1056/NEJMra1004965 22150039

[3] Lundkvist J Kastäng F Kobelt G. The burden of rheumatoid arthritis and access to treatment: Health burden and costs. *Eur J Health Econ.* (2008) 8:S49–60. 10.1007/s10198-007-0088-8 18157732

[4] Safiri S Kolahi A Hoy D Smith E Bettampadi D Mansournia M Global, regional and national burden of rheumatoid arthritis 1990-2017: A systematic analysis of the Global burden of disease study 2017. *Ann Rheum Dis.* (2019) 78:1463–71. 10.1136/annrheumdis-2019-215920 31511227

[5] Yelin E Meenan R Nevitt M Epstein W. Work disability in rheumatoid arthritis: Effects of disease, social, and work factors. *Ann Intern Med.* (1980) 93:551–6. 10.7326/0003-4819-93-4-551 7436187

[6] Brzustewicz E Henc I Daca A Szarecka M Sochocka-Bykowska M Witkowski J Autoantibodies, C-reactive protein, erythrocyte sedimentation rate and serum cytokine profiling in monitoring of early treatment. *Cent Eur J Immunol.* (2017) 42:259–68. 10.5114/ceji.2017.70968 29204090 PMC5708207

[7] Aletaha D Ramiro S. Diagnosis and management of rheumatoid arthritis. *JAMA.* (2018) 320:1360–72. 10.1001/jama.2018.13103 30285183

[8] Littlejohn E Monrad S. Early diagnosis and treatment of rheumatoid arthritis. *Prim Care Clin Off Pract.* (2018) 45:237–55. 10.1016/j.pop.2018.02.010 29759122

[9] Zinellu A Mangoni A. Neutrophil-to-lymphocyte and platelet-to-lymphocyte ratio and disease activity in rheumatoid arthritis: A systematic review and meta-analysis. *Eur J Clin Invest.* (2023) 53:e13877. 10.1111/eci.13877 36121342

[10] Liu B Wang J Li Y Li K Zhang Q. The association between systemic immune-inflammation index and rheumatoid arthritis: evidence from NHANES 1999-2018. *Arthritis Res Ther.* (2023) 25:34. 10.1186/s13075-023-03018-6 36871051 PMC9985219

[11] Sproston N Ashworth J. Role of C-reactive protein at sites of inflammation and infection. *Front Immunol.* (2018) 9:754. 10.3389/fimmu.2018.00754 29706967 PMC5908901

[12] Arroyo V García-Martinez R Salvatella X. Human serum albumin, systemic inflammation, and cirrhosis. *J Hepatol.* (2014) 61:396–407. 10.1016/j.jhep.2014.04.012 24751830

[13] Zavalaga-Zegarra H Palomino-Gutierrez J Ulloque-Badaracco J Mosquera-Rojas M Hernandez-Bustamante E Alarcon-Braga E C-reactive protein-to-albumin ratio and clinical outcomes in COVID-19 patients: A systematic review and meta-analysis. *Trop Med Infect Dis.* (2022) 7:186. 10.3390/tropicalmed7080186 36006278 PMC9414550

[14] Kim M Ahn J Song J Choi H Ann H Kim J The C-reactive protein/albumin ratio as an independent predictor of mortality in patients with severe sepsis or septic shock treated with early goal-directed therapy. *PLoS One.* (2015) 10:e0132109. 10.1371/journal.pone.0132109 26158725 PMC4497596

[15] Luo B Sun M Huo X Wang Y. Two new inflammatory markers related to the CURB-65 score for disease severity in patients with community-acquired pneumonia: The hypersensitive c-reactive protein to albumin ratio and fibrinogen to albumin ratio. *Open Life Sci.* (2021) 16:84–91. 10.1515/biol-2021-0011 33817301 PMC7874604

[16] Zhang D Yan H Wei Y Liu X Zhuang Z Dai W C-Reactive protein/albumin ratio correlates with disease severity and predicts outcome in patients with aneurysmal subarachnoid hemorrhage. *Front Neurol.* (2019) 10:1186. 10.3389/fneur.2019.01186 31781024 PMC6861440

[17] Yang W Zhang W Ying H Xu Y Zhang J Min Q Two new inflammatory markers associated with disease activity score-28 in patients with rheumatoid arthritis: Albumin to fibrinogen ratio and C-reactive protein to albumin ratio. *Int Immunopharmacol.* (2018) 62:293–8. 10.1016/j.intimp.2018.07.007 30048859

[18] Kaplan H Cengiz G Şaş S Eldemir YÖ. Is the C-reactive protein-to-albumin ratio the most remarkable simple inflammatory marker showing active disease in patients with axial spondyloarthritis, psoriatic arthritis, and rheumatoid arthritis? *Clin Rheumatol.* (2023) 42:2959–69. 10.1007/s10067-023-06703-8 37470884

[19] Loprinzi P. Dose–response association of moderate-to-vigorous physical activity with cardiovascular biomarkers and all-cause mortality: Considerations by individual sports, exercise and recreational physical activities. *Prev Med.* (2015) 81:73–7. 10.1016/j.ypmed.2015.08.014 26307435

[20] Wu Y Chen Y Yang X Chen L Yang Y. Neutrophil-to-lymphocyte ratio (NLR) and platelet-to-lymphocyte ratio (PLR) were associated with disease activity in patients with systemic lupus erythematosus. *Int Immunopharmacol.* (2016) 36:94–9. 10.1016/j.intimp.2016.04.006 27111516

[21] Gulfe A Aletaha D Saxne T Geborek P. Disease activity level, remission and response in established rheumatoid arthritis: Performance of various criteria sets in an observational cohort, treated with anti-TNF agents. *BMC Musculoskelet Disord.* (2009) 10:41. 10.1186/1471-2474-10-41 19389230 PMC2678269

[22] Ward M. Laboratory testing for systemic rheumatic diseases. *Postgrad Med.* (1998) 103:93–100. 10.3810/pgm.1998.02.310 9479309

[23] Liu X Li J Sun L Wang T Liang W. The association between neutrophil-to-lymphocyte ratio and disease activity in rheumatoid arthritis. *J Clin Lab Anal.* (2015) 30:597–601. 10.1002/jcla.21908 26666737 PMC6807125

[24] Wollheim F. Markers of disease in rheumatoid arthritis. *Curr Opin Rheumatol.* (2000) 12:200–4. 10.1097/00002281-200005000-00007 10803749

[25] Contini P Murdaca G Puppo F Negrini S. HLA-G expressing immune cells in immune mediated diseases. *Front Immunol.* (2020) 11:1613. 10.3389/fimmu.2020.01613 32983083 PMC7484697

[26] Tominaga T Nonaka T Sumida Y Hidaka S Sawai T Nagayasu T. The C-reactive protein to albumin ratio as a predictor of severe side effects of adjuvant chemotherapy in stage III colorectal cancer patients. *PLoS One.* (2016) 11:e0167967. 10.1371/journal.pone.0167967 27930703 PMC5145220

[27] Ishizuka M Nagata H Takagi K Iwasaki Y Shibuya N Kubota K. Clinical significance of the C-reactive protein to albumin ratio for survival after surgery for colorectal cancer. *Ann Surg Oncol.* (2016) 23:900–7. 10.1245/s10434-015-4948-7 26530445

[28] Strang F Schunkert H. C-reactive protein and coronary heart disease: All said–is not it? *Mediators Inflamm.* (2014) 2014:757123. 10.1155/2014/757123 24808639 PMC3997990

[29] Caraceni P Tufoni M Bonavita M. Clinical use of albumin. *Blood Transfus.* (2013) 11:s18–25. 10.2450/2013.005s 24333308 PMC3853979

[30] Gupta D Lis C. Pretreatment serum albumin as a predictor of cancer survival: A systematic review of the epidemiological literature. *Nutr J.* (2010) 9:69. 10.1186/1475-2891-9-69 21176210 PMC3019132

[31] Caraceni P Tufoni M Zaccherini G Riggio O Angeli P Alessandria C. On-treatment serum albumin level can guide long-term treatment in patients with cirrhosis and uncomplicated ascites. *J Hepatol.* (2021) 74:340–9. 10.1016/j.jhep.2020.08.021 32853747

[32] Bai M Ji Z Wang S Lin Z Pan S Huang K Prognostic value of C-reactive protein/albumin ratio in neurocritically Ill patients. *Minerva Anestesiol.* (2019) 85:1299–307. 10.23736/S0375-9393.19.13625-5 31486619

[33] Liu Y Chen S Zheng C Ding M Zhang L Wang L The prognostic value of the preoperative C-reactive protein/albumin ratio in ovarian cancer. *BMC Cancer.* (2017) 17:285. 10.1186/s12885-017-3220-x 28431566 PMC5399817

[34] You J He Y Xu M Qian M. Association between the C-reactive protein to albumin ratio with asthma and mortality in adult: A population-based study. *Sci Rep.* (2024) 14:20573. 10.1038/s41598-024-71754-z 39232083 PMC11375090

[35] Ranzani O Zampieri F Forte D Azevedo L Park M. C-reactive protein/albumin ratio predicts 90-day mortality of septic patients. *PLoS One.* (2013) 8:e59321. 10.1371/journal.pone.0059321 23555017 PMC3595283

[36] Sheinenzon A Shehadeh M Michelis R Shaoul E Ronen O. Serum albumin levels and inflammation. *Int J Biol Macromol.* (2021) 184:857–62. 10.1016/j.ijbiomac.2021.06.140 34181998

